# EIT guided evaluation of regional ventilation distributions in neonatal and pediatric ARDS: a prospective feasibility study

**DOI:** 10.1186/s12931-025-03134-8

**Published:** 2025-02-19

**Authors:** Leon Soltész, Judith Leyens, Marieke Vogel, Thomas Muders, Christian Putensen, Florian Kipfmueller, Till Dresbach, Andreas Mueller, Lukas Schroeder

**Affiliations:** 1 Department of Neonatology and Pediatric Intensive Care, University Children´s Hospital Bonn, Bonn, Germany; 2 Division of Neonatology, Department of Pediatric and Adolescent Medicine, University Hospital RWTH Aachen, Aachen, Germany; 3 Division of Neonatology, Department of Pediatrics, BC Women’s and Children’s Hospital, University of British Columbia, Vancouver, Canada; 4 Department of Anesthesiology and Operative Intensive Care Medicine, University Hospital Bonn, Bonn, Germany

**Keywords:** Electrical impedance tomography, Regional ventilation, Neonatal, Pediatric, Acute respiratory distress syndrome

## Abstract

**Background:**

Despite international guidelines for lung protective ventilation in neonatal or pediatric acute respiratory distress syndrome (nARDS/ pARDS), prospective data on bedside monitoring tools for regional ventilation distribution and lung mechanics are still rare. As a bedside and radiation-free procedure, electrical impedance tomography (EIT) offers a practical and safe approach for analyzing regional ventilation distributions. Recent trials in adults have shown the efficacy of an individualized EIT guided strategy for the improvement of ventilator induced lung injury (VILI).

**Methods:**

We performed a single-center prospective feasibility study from November/2021 to December/2023 in the department of neonatal and pediatric intensive care medicine at the University Children´s Hospital in Bonn. All patients with diagnosis of nARDS (or history of perinatal lung disease-PLD)/ pARDS were screened for study inclusion. In all patients a decremental PEEP (positive end-expiratory pressure) trial was performed with a continuous EIT monitoring for an individual analysis of the EIT guided pixel compliance (C_EIT_) and PEEP finding (EIT-PEEP). In the offline analysis, further EIT derived indices, such as global inhomogeneity index (GI), and center of ventilation (CoV), were calculated.

**Results:**

Overall, 40 EIT measurements were performed in 26 neonatal and pediatric patients (nARDS/PLD, *n* = 6; and pARDS, *n* = 20) within a predefined decremental PEEP trial. Thirteen patients were classified as having severe nARDS (PLD)/ pARDS with an Oxygen Saturation Index (OSI) > 12 or Oxygenation Index (OI) > 16. In-hospital mortality rate was 27% in the overall cohort. The median EIT-PEEP (11mbar) was calculated as lowest, as compared to the clinically set PEEP (11.5mbar, *p* < 0.001), and the ARDSnetwork PEEP table recommendation (ARDSnet-PEEP, 14mbar, *p* = 0.018). In patients with nARDS/PLD, the EIT-PEEP was calculated 3mbar below the clinically set PEEP (*p* = 0.058) and 11 mbar below the ARDSnet-PEEP (*p* = 0.01). In the linear regression analysis, EIT-PEEP and the dynamic compliance (C_DYN_) at -2mbar presented a significant correlation with a Cohen´s R^2^ of 0.265 (β: 0.886, *p* = 0.005).

**Conclusion:**

EIT is feasible and can be performed safely in patients with diagnosis of nARDS/PLD and pARDS, even during ongoing extracorporeal membrane oxygenation (ECMO) support. An individualized PEEP finding strategy according to the EIT compliance might optimize regional ventilation distribution in these patients and can potentially decrease VILI.

**Clinical trial registration:**

The study was registered at the German Clinical Trials Register (GCT; trial number: DRKS 00034905, Registration Date 15.08.2024). The registration was performed retrospectively after inclusion of the last patient.

## Introduction

The mortality of pediatric acute respiratory distress syndrome (pARDS) is reported as 30% and can be as high as 50% in severe pARDS [1]. The incidence of pARDS has previously been 3.5/100,000 per year (Berlin definition), however, the new pARDS definition of the Pediatric Acute Lung Injury Consensus Conference (PALICC) allows approximately [2] 40% more children to be identified, so that the actual number is correspondingly higher [1, 3]. The reported prevalence for neonatal ARDS (nARDS) is 1–2%, with a mortality rate around 25%, which is comparable to the data from children suffering from pARDS [4].

Despite intensive research, development of consensus guidelines, and enhanced treatment strategies such as extracorporeal membrane oxygenation (ECMO), there is still a need for improvement of treatment strategies. The clinical picture of nARDS and pARDS is very similar and both entities are determined by various etiologies. Infections with a septic course, traumatic lung events, as well as endogenous causes have been identified as major contributors of lung damage [3–6]. Due to these heterogenous causes, an individual therapeutic approach seems to be reasonable. One of the major challenges in nARDS and pARDS is to prevent patients from ventilator induced lung injury (VILI). VILI can be origin and consequence of ARDS, and is one of the major contributors to patient’s outcome [7]. Therefore, international treatment guidelines have defined strategies for lung protective ventilation in nARDS/ pARDS (tidal volume 6-8 ml/kg, peak ventilation pressure < 30mbar, high PEEP, permissive hypercapnia) [8, 9]. There is still a lack of prospective data on the optimal PEEP level and effect of PEEP on lung mechanics and outcome, but recent studies and meta-analyses have shown a huge variability in clinically set PEEP and negative effects of high PEEPs in mild and severe pARDS [10]. Being aware of these challenges, the renewed PALICC guidelines (PALICC-2) call for prospective studies to evaluate monitoring tools for mechanical ventilation in children [8]. In clinical routine, EIT is already present in adults, but in children this technique is just part of recommendations for further investigation. Experimental and clinical data confirm its benefits in the management of children receiving mechanical ventilation by visualizing and evaluating regional ventilation changes, alveolar collapse, alveolar recruitment and lung overdistension [11]. In recent years, there has been upcoming research evaluating an individualized approach using EIT to optimize ventilator and PEEP settings in critically ill patients with ARDS, with the aim the reduce VILI and improve patient outcome [12–14]. Unfortunately, comparable data from neonatal or pediatric cohorts are still lacking. The present study aims to provide preliminary data to the existing knowledge about feasibility and benefits of EIT guidance in nARDS and pARDS. By evaluating regional compliance changes and ventilation distributions, we aim to validate how EIT can be integrated in daily clinical practice for an individualized PEEP finding and decision-making of ventilator settings in patients with nARDS and pARDS.

## Methods

### Study cohort and ethical approval

All patients admitted to the neonatal and pediatric intensive care unit (NICU/ PICU) of the University Children´s Hospital Bonn between November 2021 and December 2023 were screened for study inclusion. Inclusion criteria were: nARDS/ pARDS by PALICC-2, Montreux definition, or history of perinatal lung disease (PLD, including infants with congenital lung disease, e.g. congenital diaphragmatic hernia [CDH]) [8, 15]; need for prolonged invasive mechanical ventilation (> 24 h); bodyweight ≥ 3.5 kg, and thorax circumference ≥ 36 cm. Exclusion criteria were defined as follows: patients undergoing palliative care, single lung physiology, unstable hemodynamical or clinical condition impeding an EIT measurement, contraindication for EIT measurements according to device certification (Fa. Draeger, e.g. implanted pacemaker). The methods used for the clinical research were performed in accordance with the STROBE (strengthening the reporting of observational studies in epidemiology) guidelines [16] and in accordance with the Declaration of Helsinki.

Prior to study inclusion, written informed consent was obtained by the legal guardians of the patients. Ethical approval was given by the Institutional Review Board of the Medical Center of the University of Bonn (local running number 048/21). The study was retrospectively registered at the German Clinical Trials Register (GCT; trial number: DRKS 00034905, Registration date 15.08.2024) after finalization of the study and inclusion of the last patient.

### Monitoring and ventilation data

EIT was performed regardless of other organ failures, genetic defects, or ARDS origin. Vital signs, ventilation data and ventilator settings were continuously collected by the in-house electronic documentation system (Integrated Care Manager-ICM by Dräger Medical GmbH). Due to the use of three different ventilators in our department (Dräger [Babylog VN500 and Evita V500] and Maquet [Servo N]), the dynamic compliance (C_DYN_) was calculated (${C}_{dyn}=\frac{\text{tidal}\,\text{volume}}{\text{driving}\,\text{pressure}}$) when not recorded by the ICM. Analyzed ventilation parameters are shown in Table 1. Arterial blood gas (ABG) values were obtained in the unit using the Rapidlab 1200 system (Siemens Healthcare, Erlangen Germany).

**Table 1:** Analyzed ventilation related parameters

Parameters
Ventilation mode
Fraction of inspired oxygen (FiO_2_)
Use of nitric oxide (NO)/ isoflurane
Positive end-expiratory pressure (PEEP)
Clinically set PEEP (as set by the physician)
EIT-PEEP (according to the Costa approach)
ARDSnet-PEEP (according to FiO_2_/PEEP table from ARDSnet recommendation)
Positive inspiratory pressure (PIP)
Mean airway pressure (MAP)
Driving pressure (∆P)
Respiratory rate (RR, as set at the respirator and spontaneous RR by the patient)
Tidalvolume (V_T_, inspiratory and expiratory)
Dynamic compliance (C_DYN_)
Oxygen saturation index (OSI)
Inspiratory time (T_I_)
Arterial blood gas analysis (ABG: paO_2_, paCO_2_, SaO_2_)

### EIT measurement and EIT indices

After admission to the NICU or PICU and after informed written consent of the legal parental guardians, a standardized PEEP titration and decremental PEEP trial was performed as early as possible after starting invasive pressure-controlled mechanical ventilation and meeting criteria of nARDS or pARDS (T1). When possible, EIT measurements were repeated in each individual patient (T2, T3,.). The EIT measurements (frame rate 50 Hz) were performed using the PulmoVista 500 EIT device (Fa. Draeger, Lübeck, Germany) and the corresponding 16-electrode silicone-belt. The electrode belt was applied around the thorax at the nipple-level and in newborns and infants < 10 kg directly under the axilla level. The PEEP titration was interrupted immediately when patient’s clinical situation deteriorated (e.g., desaturation or severe hypotension). PulmoVista500 software and custom-made software (Matlab 21a; The MathWorks Inc.) were used for online and offline EIT indices evaluation. Primarily, the EIT pixel compliance (C_EIT_) was calculated based on the formula $\:{C}_{PIXEL}=\frac{\varDelta\:\text{Z}}{Pplateau-PEEP}$, as described by Costa et al. [17]. This calculation was used to explore the EIT-PEEP (mbar), which is defined as the PEEP at the crossing-point where the cumulated overdistension (OD) and lung collapse (LC; both as percentage of lung area) is at the minimum. In addition, the center of ventilation (CoV) describes the horizontal (right to left [CoV_X_], 0–1) and vertical (ventral to dorsal [CoV_Y,_ 0–1]) spatial distribution of lung ventilation [18, 19]. Integration of the distribution of tidal impedance variations of all EIT pixels is represented by the global inhomogeneity (GI) index in a single numeric value [18, 20]. Furthermore, the ventilated lung area was calculated during the different PEEP levels in 4 predefined regions of interest (ROIs: ventral, mid-ventral, mid-dorsal, dorsal).

### Standardized decremental PEEP titration

Clinically set PEEP, as set by the attending physician, was defined as baseline PEEP (BP, mbar). Clinically set PEEP was adjusted by the physician according to the in-house clinically routine and considering the different points: (a) chest x-rays imaging and lung ultrasound (position of the diaphragm and lung opacities/ lung overdistension), (b) ABG values with focus on the of partial pressure of carbon dioxide (paCO_2;_ normal to permissive hypercarbia with tolerated pH of 7.25–7.40), and partial pressure of oxygen (paO_2_), (c) dynamic compliance, driving pressure (targeted range < 15mbar), and tidal volumes (targeted range 4-6 ml/kg/ predicted body weight) as measured by the ventilator, and (d) cardiac function, evidence of pulmonary hypertension and preload situation. Starting from BP, the PEEP was increased + 4mbar and then decreased in 2 mbar steps every 5 min (PEEP titration: BP + 4mbar, BP + 2mbar, BP + 0mbar, BP – 2mbar, BP -4mbar), with a constant driving pressure (∆P) and fraction of inspired oxygen (FiO_2_). At the end of each PEEP level an ABG was obtained from an indwelling arterial line for calculation paO_2_, paCO_2_.

### Group stratification, statistics and outcome measures

The primary outcome factor was identification of the EIT-PEEP according to the EIT derived compliance (C_EIT_). The EIT-PEEP was then compared with the clinically set PEEP as set by the attending physician and the lower table of the ARDSnet protocol recommendation (ARDSnet-PEEP) [21, 22]. The total cohort was then subdivided into patients with pARDS (group A), and patients with nARDS and history of PLD (group B) for further subgroup analysis.

Secondary outcome measures were further EIT derived indices as GI and CoV as well as identification of the optimal ABG values during PEEP titration (paO_2_, pCO_2_, SaO_2_), and identification of the best respirator derived compliance (C_dyn_) during PEEP titration. Furthermore, the duration of mechanical ventilation and in-hospital mortality were defined as secondary outcome measures. We did not perform a power-analysis, as there is a lack of comparable data, and the feasibility study was conducted as basis for a future longitudinal randomized control trial.

Statistical calculations were analyzed using IBM SPSS Version 29.0.2.0. Descriptive data is presented as absolute numbers (n) and percentage. Non-normally distributed data are presented as median with interquartile range (IQR). Repeated measurements over different PEEP levels were calculated using ANOVA with repeated measurements and Bonferroni-adjusted post-hoc analysis. For comparison between subgroups and timepoints (non-normally distributed, continuous variables), a Mann-Whitney U test or Wilcoxon test was performed. For categorical variables, the Pearson’s Chi^2^ test and Fisher’s exact test were applied, as appropriate. P-values below 0.05 were considered as statistically significant.

## Results

**Fig. 1:**
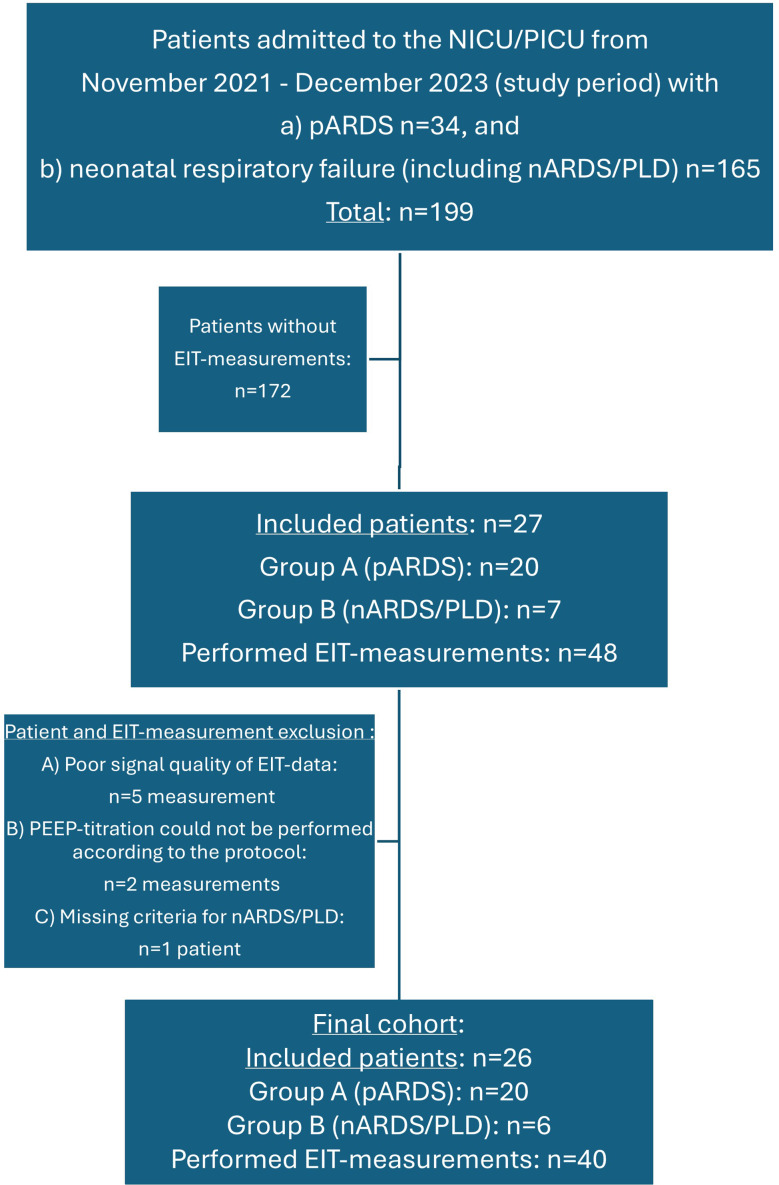
CONSORT flow-chart of the study population and patients admitted to our neonatal and pediatric intensive care unit with the diagnosis of nARDS/ history of PLD and pediatric ARDS. Patients with pARDS were referred to the ICD diagnosis J80.0 = acute respiratory distress syndrome (ARDS): between 28 days and 18 years [J80.0 [1–9] = subgroups and severity of ARDS] and patients with neonatal respiratory failure were referred to the ICD diagnosis P28.5 = respiratory failure in neonates. Abbreviations: EIT: electrical impedance tomography; nARDS: neonatal acute respiratory distress syndrome; NICU: neonatal intensive care unit; pARDS: pediatric acute respiratory distress syndrome; PICU: pediatric intensive care unit: PLD: perinatal lung disease

The CONSORT Flow-chart is displayed in Fig. 1. Overall, 26 patients were prospectively enrolled and included in the study, with overall 47 EIT measurements performed in these patients. Seven EIT measurements were excluded from the final statistical analysis due to an incomplete performance of the PEEP titration protocol (*n* = 2) or poor quality of the EIT data set, which could not be analyzed appropriately (*n* = 5; inadequate EIT signal quality and inability to detect single breathes properly). All patients’ characteristics and epidemiological data are displayed in Table 2. 77% of patients with pARDS were allocated to group A (*n* = 20) and 23% of patients with nARDS/history of PLD to group B (*n* = 6). More than 50% of patients in both groups were classified as severe ARDS (OSI > 12 or OI > 16). More detailed information about the suspected cause of ARDS and outcome data are illustrated also in Table 2.

**Table 2:** Data are presented as absolute number (n) with % or median with IQR (25/75) for non-normally distributed data. Any organ failure was confirmed by state-of-the-art diagnostic. Brain (incl. structural, ischemic, hemorrhagic lesions), heart (incl. only structural heart defects confirmed by echocardiography), liver failure (liver cirrhosis), renal failure (incl. structural, tumor and infectious disease, acute kidney failure in patient history not related to recent ARDS), genetics (incl. only genetically confirmed aberrations; trisomy 21, 8 mosaic, ABCA3 frameshift mutation and VACTERL). Abbreviations: nARDS: neonatal acute respiratory distress syndrome, pARDS: pediatric acute respiratory distress syndrome, ECMO = extracorporeal membrane oxygenation, PALICC-2 = Pediatric Acute Lung Injury Consensus Conference, PLD = perinatal lung disease, T1 = first EIT measurement as soon as possible after admission and start of mechanical ventilation

Epidemiological Data (*N* = 26 patients)				
Age, years	2 (0.6/6.8)	**Organ failure (not associated with ARDS)**,** n (%)**		
Female sex, n (%)	16 (62)	*Brain*		7 (27)
Body weight, kg	10 (6/25)	*Major cardiac defect*		11 (42)
Thoracic circumference, cm	52 (42/71)	*Liver failure*		1 (4)
T1 in days after admission, d	3.5 (1/26)	*Renal failure*		7 (27)
**EIT measurements**,** n (%)**		**Genetics**,** n (%)**		
*Overall measurements*	40	*Trisomy 21*		4 (15)
*One measurement*,* n (%)*	17 (65)	*Other aberration*		2 (8)
*≥Two measurements*,* n (%)*	9 (35)	Veno-venous ECMO, n (%)		10 (39)
*Under ECMO support*,* n (%)*	6 (23)	ECMO duration, d		14 (5/56)
**Main diagnosis**,** n (%)**		In-hospital length of stay, d		44 (15/79)
*pARDS (PALICC-2)*	20 (77)	Duration of mechanical ventilation, d		21 (7/55)
*nARDS/PLD (Montreux)*	6 (23)	Days of oxygen supply, d		11 (25/74)
*severe pARDS*	10 (50)	In-hospital mortality, n (%)		7 (27)
*severe nARDS*	4 (67)			
*severe ARDS*,* overall*	14 (54)			
**Pathogen detection**,** n (%)** (blood stream infection or respiratory tract)				
*No pathogen found*	8 (31)			
*Bacterial*	3 (12)			
*Viral*	12 (46)			
*Covid-19*	5 (19)			
*Combined*	3 (12)			

### Ventilation and respirator data

All patients were on invasive mechanical ventilation. Ventilator settings and modes are displayed in Table 3. At the time of PEEP titration and EIT measurements (*n* = 40), 60% (*n* = 23) of the examinations were performed during a pressure-controlled biphasic positive airway pressure (PC-BIPAP) mode and 40% (*n* = 15) during a pressure-controlled synchronized intermittent mandatory ventilation (PC-SIMV) mode. Two EIT measurements were performed during a pressure-supported spontaneous continuous positive airway pressure (SPN-CPAP) mode. All patients were treated with midazolam or inhaled sevoflurane (50% of patients) as a sedative, in addition to fentanyl or remifentanil. A neuromuscular blockade was used (cisatracurium or vecuroniumbromid) only if needed and patients were uncomfortable on assisted ventilation mode. In two-thirds of the EIT measurements patients were treated with inhaled nitric oxide (iNO).

**Table 3:** Ventilator settings. Data are presented as median with IQR (25/75). Abbreviations: ARDSNet-PEEP = PEEP following the ARDSnet recommendation table [low PEEP/FiO_2_], clinically set PEEP = PEEP as set by the attending physician, EIT-PEEP = PEEP level according to the crossing-point of the curves for the estimated lung overdistension and lung collapse according to the EIT-pixel compliance (Costa et al.), MAP = mean airway pressure, OSI = oxygenation saturation index, PEEP = positive end expiratory pressure, PIP = peak-inspiratory-pressure, ΔP = driving pressure, RR = respiratory rate

Parameters EIT Measurements (*N* = 40)			
	Median	IQR (25/75)	
FiO_2_, %	87.5	55	100
OSI	15	9.4	20
Clinically set PEEP, mbar	11.5	10	15
ARDSNet-PEEP, mbar	14	10	18
EIT-PEEP (Costa et al.), mbar	11	8.5	14
PIP, mbar	26	22	30
ΔP, mbar	13	11	17
MAP, mbar	18	15	22
RR, breath/min	25	20	30
RR incl. spontaneous breathing	28	20	37
Inspiratory time, s	1	0,8	1.2

### Feasibility and safety of EIT data recording

Valid EIT data of sufficient quality were obtained in 40/48 (83%) EIT measurements. Seven examinations (17.5%) could not be completed in their intended range. In five patients (12.5%) the last PEEP titration step (4mbar below BP) resulted in a subsequent desaturation (SpO_2_ < 90%), with a suspected lung de-recruitment of dorsal gravity-dependent lung regions. In two of these patients the PEEP titration needs to be interrupted directly (SpO_2_ decreasing < 80%), resulting in an exclusion of these two patients (see flow-chart, Fig. 1). In the other patients SpO_2_ improved rapidly after the end of the PEEP titration when adjusting PEEP towards baseline. No EIT measurement need be discontinued due to hemodynamic instability (severely decreased blood pressure). In five patients EIT data sets were of poor signal quality and could not be analyzed (see flow-chart, Fig. 1).

### PEEP titration, EIT measurements

**Fig. 2:**
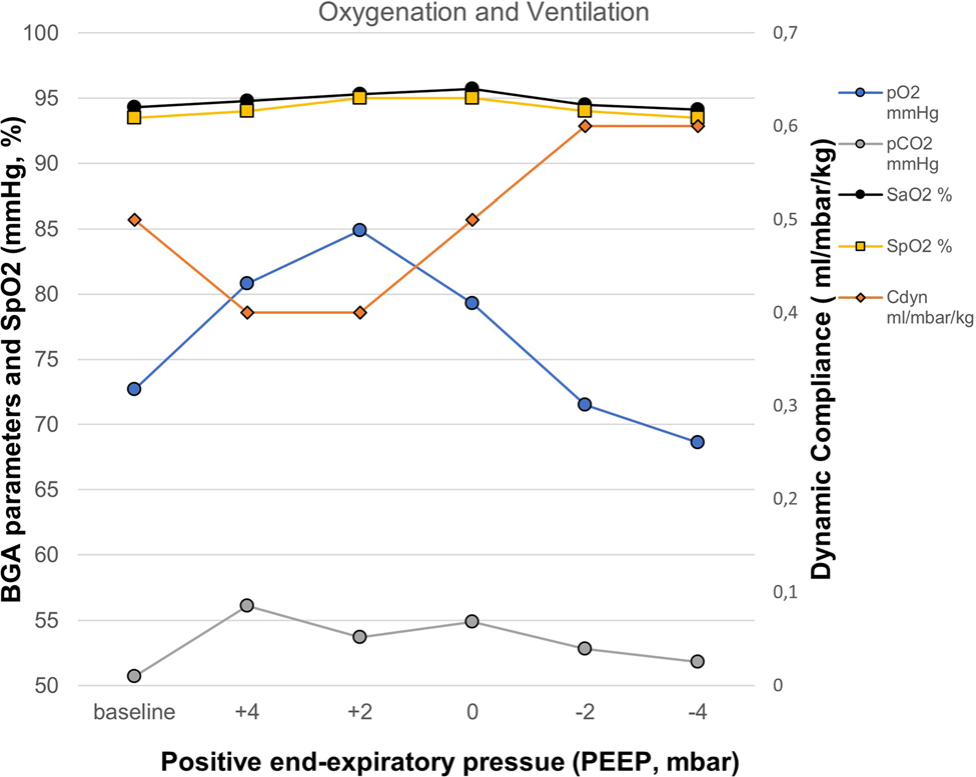
Blood gas analysis with paO_2_- and paCO_2_-measurements, as well as SpO_2_-, SaO_2_-, and dynamic compliance measurements during the decremental PEEP titration. Abbreviations: BGA: blood gas analysis; paO_2_: partial arterial pressure of oxygen; paCO_2_: partial arterial pressure of carbon dioxide; SaO_2_: arterial oxygen saturation; SpO_2_: pulse-oxymetric oxygen saturation

**Fig. 3:**
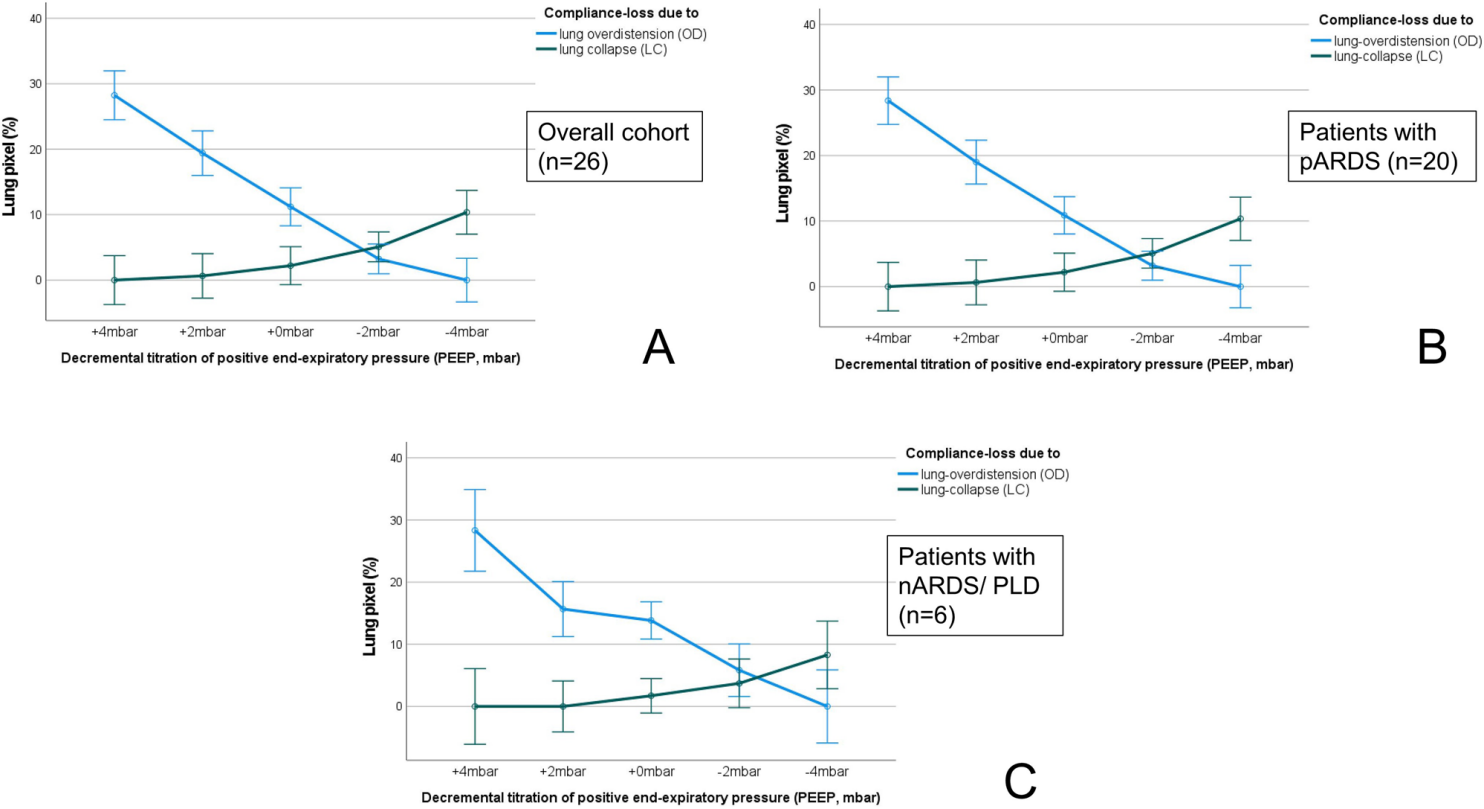
Compliance loss due to overdistension (OD, blue line) and lung collapse (LC, green line) during the decremental PEEP titration for the overall cohort (**A**), for subgroup A (pARDS) (**B**), and subgroup B (nARDS/PLD). The crossing-point between both lines with the lowest estimated lung overdistension and lung collapse according to the EIT pixel compliance (Costa et al.) represents the optimal EIT based PEEP level. Abbreviations: nARDS: neonatal acute respiratory distress syndrome, pARDS: pediatric acute respiratory distress syndrome, PLD: perinatal lung disease

**Fig. 4:**
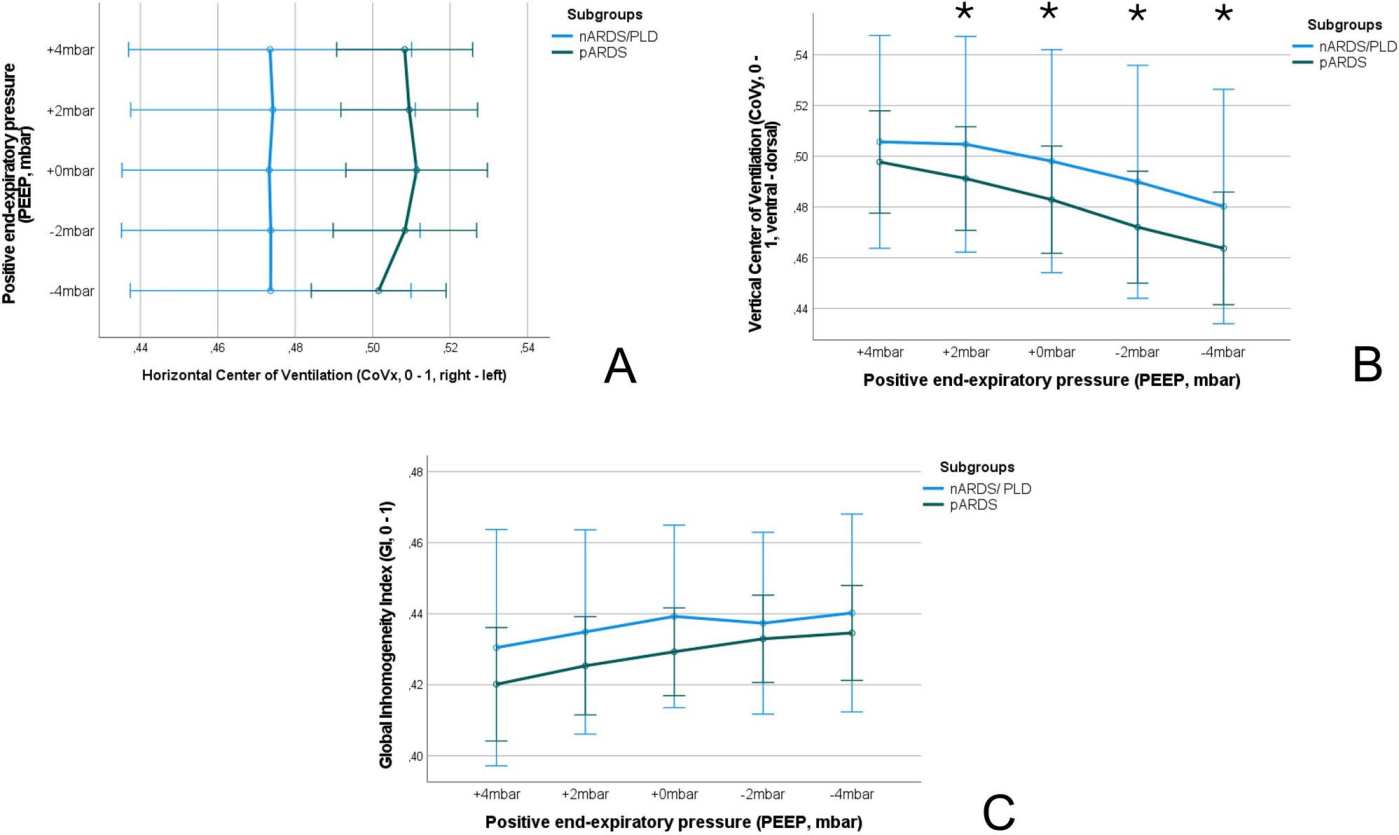
The horizontal and vertical Center of Ventilation (CoV_X_, CoV_Y,_**A** and **B**), and the Global Inhomogeneity Index (GI, **C**) for the respective subgroups (nARDS/PLD vs. pARDS) during the decremental PEEP titration are displayed. The asterisk is illustrating a p-value < 0.05 when comparing the respective value with the value calculated at PEEP level + 4mbar. Abbreviations: nARDS: neonatal acute respiratory distress syndrome, pARDS: pediatric acute respiratory distress syndrome, PLD: perinatal lung disease

**Fig. 5:**
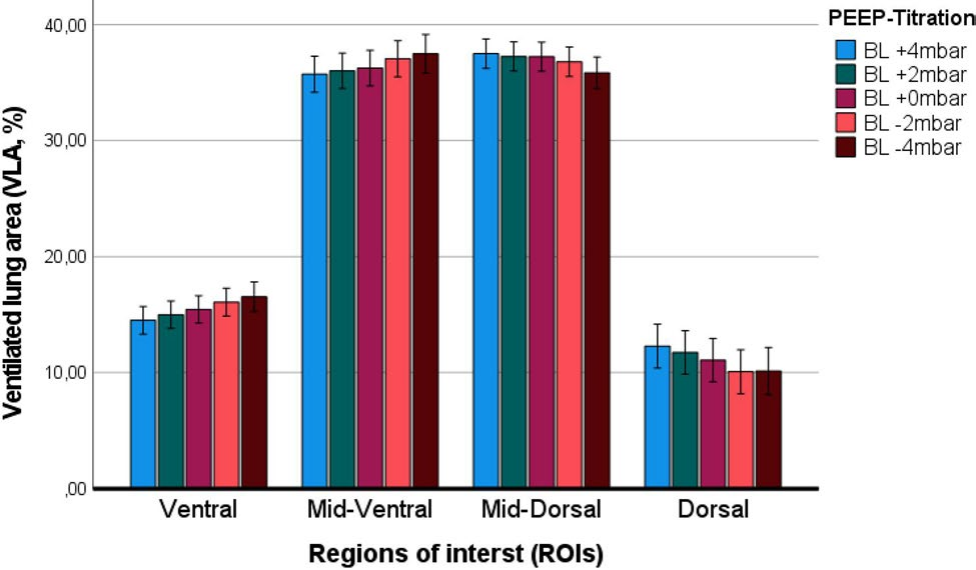
The ventilated lung area (bars are presented with 95% CI), separated into 4 regions of interest (ventral, mid-ventral, mid-dorsal, dorsal) during the PEEP titration maneuver. Abbreviations: BL: baseline

The evaluation of the ventilator derived dynamic compliance (C_DYN_) measurements during PEEP titration is illustrated and ABG values as well as saturation values are displayed in Fig. 2. In all measurements the best C_DYN_ was detected at a PEEP of -2mbar below the clinically set PEEP. When comparing both subgroups (A vs. B) best C_DYN_ was detected at a PEEP − 2mbar below the clinically set PEEP, with an overall gain of C_DYN_ around 20% (*p* = 0.065) (see Fig. 2, and Table 4). The paO_2_ and paCO_2_ almost remained unaffected and showed no significant decrease or increase (*p* > 0.05), even when lowering the PEEP − 4mbar. The calculation for OD and LC with the detection of the crossing-point of both curves, the illustration of the GI, CoV_X_ and CoV_Y_, as well as the PEEP calculation according to the different methods (clinically set PEEP, EIT-PEEP, ARDSnet-PEEP) are illustrated in Figs. 3, 4 and 5(A-C).

**Table 4:** All data are presented as median with IQR (25/75). Percentage change (∆) refers to baseline compared with the according PEEP level. Abbreviations: C_dyn_ = dynamic compliance recorded by the ventilator or calculated $\:{\text{C}}_{\text{d}\text{y}\text{n}\:=}\frac{\text{T}\text{i}\text{d}\text{a}\text{l}\,\text{v}\text{o}\text{l}\text{u}\text{m}\text{e}}{\text{D}\text{r}\text{i}\text{v}\text{i}\text{n}\text{g}\text{p}\text{r}\text{e}\text{s}\text{s}\text{u}\text{e}}$, TVi = inspiratory tidal volume, TVe = expiratory tidal volume

PEEP Titration	C_dyn_ (ml/mbar/kg)	∆%	TVi (ml/kg)	∆%	TVe (ml/kg)	∆%
*BP + 4mbar*	*29 (16.5–35)*	*0*	*0.497 (0.479–0.535)*		*0.5 (0.463–0.543)*	
BP + 2mbar	18 (8-24.25)	0	0.496 (0.479–0.533)	-0.2	0.495 (0.454–0.533)	-1
BP + 0mbar	11 (3-14.25)	1 (0-3.25)	0.499 (0.480–0.534)	0,4	0.489 (0.450–0.522)	-2.2
BP -2 mbar	2 (0–5)	3 (0-8.25)	0.497 (0.479–0.529)	0	0.481 (0.436–0.515)	-3.8
BP -4 mbar	0	6 (2-11.5)	0.493 (0.471–0.529)	-0.8	0.467 (0.418–0.504)	-6.6

**Fig. 6:**
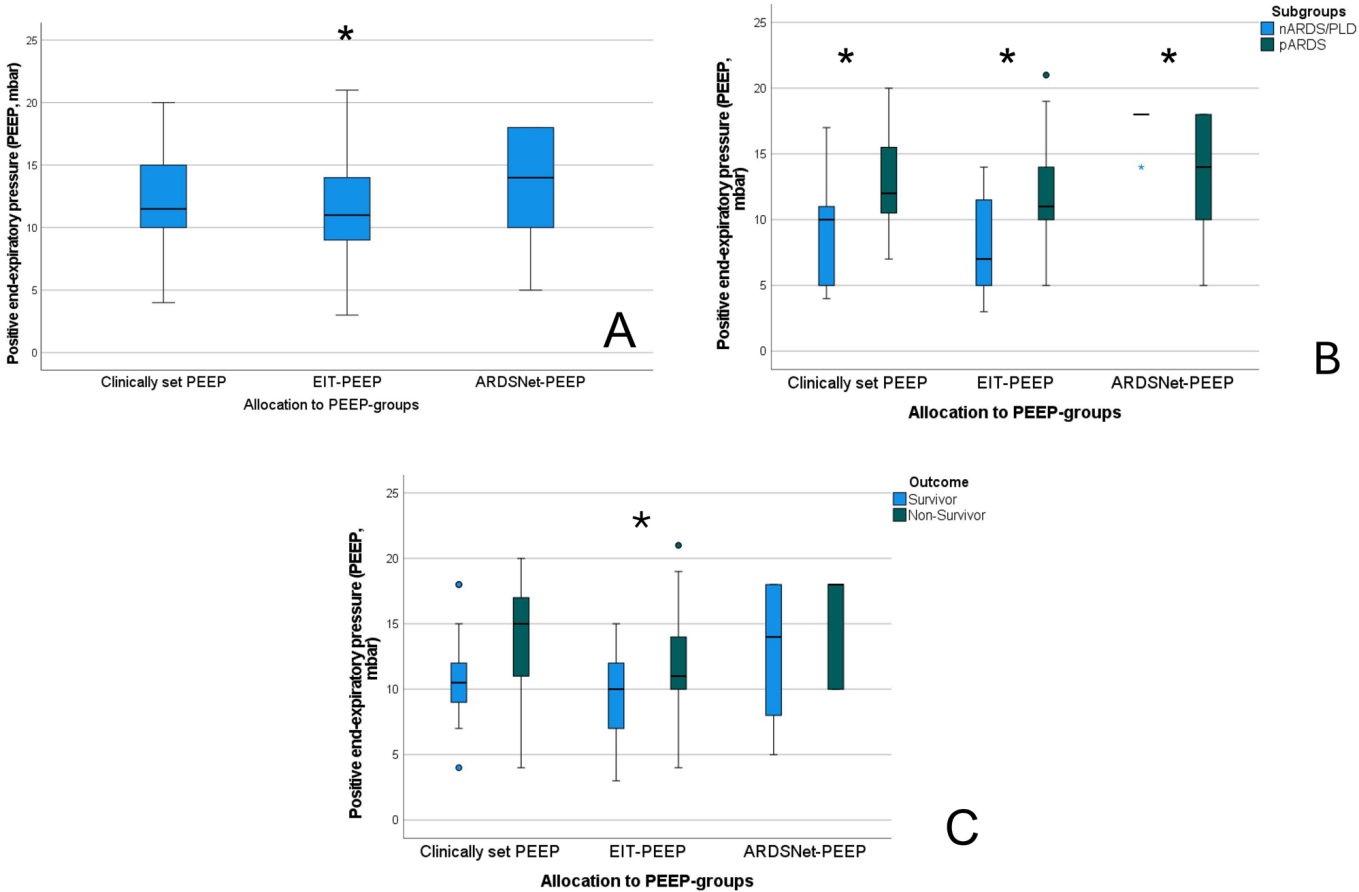
Calculation of the median values for the clinically set PEEP, the EIT-PEEP, and the ARDSnet-PEEP in the overall cohort (**A**), for the respective subgroups (**B**), and according to patient’s outcome (**C**). The asterisk is illustrating a p-level < 0.05 for comparison of EIT-PEEP compared to the clinically set PEEP (**A**) or comparison between subgroups (**B** and **C**). Abbreviations: nARDS: neonatal acute respiratory distress syndrome, pARDS: pediatric acute respiratory distress syndrome, PLD: perinatal lung disease

Overall, at a PEEP of -2mbar compared to the clinically set PEEP, the cumulated OD and LC (C_EIT_) were both defined as mild/tolerable (≤ 5%), defining the best C_EIT_ and the optimal crossing-point between both data sets (OD vs. LC; compare Fig. 3A-C, and Table 5). There were no differences found between patients from group A or group B regarding findings for OD and LC (C_EIT_). The GI and CoV_X_ remained stable when lowering PEEP from + 4mbar to -2mbar, without significant difference between PEEP levels and subgroups. However, the CoV_Y_ decreased significantly when lowering PEEP level towards − 4mbar, but without significant difference between subgroups (Fig. 5B). When lowering the PEEP to -4mbar the LC (+ 6%, *p* < 0.001, Fig. 2A), and the CoV_Y_ (-0.03, *p* < 0.001, Fig. 3B) changed significantly, might indicating a loss of ventilated lung area. The ventilated lung area during the PEEP titration is illustrated in Fig. 4. When comparing the clinically set PEEP, the EIT-PEEP, and ARDSnet-PEEP (Fig. 6A-C) of the overall cohort, the EIT-PEEP was calculated − 0.5mbar below the clinically set PEEP (*p* < 0.001, median clinically set PEEP 11.5 vs. 11 EIT-PEEP), and about − 2.5mbar below the ARDSnet-PEEP recommendation (low FiO_2_/PEEP table, *p* = 0.018). PEEP levels (clinically set PEEP, EIT-PEEP, and ARDSnet-PEEP) between subgroups (nARDS/PLD vs. pARDS) differed significantly (*p* = 0.034, *p* = 0.021, and *p* = 0.024, respectively). When separating only for patients from group A, EIT-PEEP differed significantly from clinically set PEEP (*p* < 0.001), but did not differ from ARDSnet-PEEP (*p* = 0.470). When looking for patients allocated to group B, EIT-PEEP was calculated − 3mbar below the clinically set PEEP (*p* = 0.063) and − 9mbar below the ARDSnet-PEEP recommendation (*p* = 0.008). In patients with fatal outcome (in-hospital mortality), EIT-PEEP levels between survivors and non-survivors differed significantly (*p* = 0.009), with higher EIT-PEEP values in patients with fatal outcome. PEEP levels for clinically set PEEP and ARDSnet-PEEP tended to be higher in patients with fatal outcome, but without significant difference (clinically set PEEP: *p* = 0.110; ARDSnet-PEEP: *p* = 0.053). In the linear regression analysis, the EIT-PEEP (with best C_EIT_) and C_DYN_ at -2mbar presented the best correlation with a Cohen´s R^2^ of 0.265 (β: 0.886, *p* = 0.005), as when compared to clinically set PEEP (β=-0.810, *p* = 0.010) and ARDSnet-PEEP (β:0.289, *p* = 0.060).

**Table 5:** All data are presented as median with IQR (25/75). Percentage change (∆) refers to baseline compared with the according PEEP level. Abbreviations: COV = center of ventilation (x = horizontal change, 0–1 = right-left; y = vertical change, 0–1 = ventral-dorsal); GI = global Inhomogeneity Index; LC = EIT derived compliance-loss (%) due to lung collapse (LC); OD = EIT derived compliance-loss (%) due to overdistension (OD)

PEEP Titration	OD	LC	COV_X_	∆ %	COV_Y_	∆ %
*BP + 4mbar*	*29 (16.5–35)*	*0*	*0.497 (0.479–0.535)*		*0.5 (0.463–0.543)*	
BP + 2mbar	18 (8-24.25)	0	0.496 (0.479–0.533)	-0.2	0.495 (0.454–0.533)	-1
BP + 0mbar	11 (3-14.25)	1 (0-3.25)	0.499 (0.480–0.534)	+ 0,4	0.489 (0.450–0.522)	-2.2
BP -2 mbar	2 (0–5)	3 (0-8.25)	0.497 (0.479–0.529)	0	0.481 (0.436–0.515)	-3.8
BP -4 mbar	0	6 (2-11.5)	0.493 (0.471–0.529)	-0.8	0.467 (0.418–0.504)	-6.6

## Discussion

The key findings of the present study are as follows: EIT guided PEEP titrations are feasible in both, patients with nARDS/PLD and pARDS, and even in patients with low body weights (> 3.5 kg). The EIT-PEEP was calculated significantly lower than the clinically set PEEP and the ARDSnet-PEEP in patients with pARDS. When subclassifying patients with nARDS/PLD, clinically set PEEP and EIT-PEEP did not differ significantly, whereas EIT-PEEP and ARDSnet-PEEP differed markedly. In all patients, and confirmed for both subgroups, the C_DYN_ increased markedly when lowering the clinically set PEEP towards the EIT-PEEP (highest C_EIT_, crossing-point of lowest OD and lowest LC). The C_DYN_ at -2mbar correlated significantly with the EIT-PEEP (β = 0.886, *p* = 0.005). Nevertheless, when PEEP level decreases towards − 4mbar, the CoV_Y_ decreases simultaneously, which on the one hand might indicate a de-recruitment of gravity-dependent lung areas or on the other hand indicate a deflation of overdistended ventral lung regions, resulting in a ventral shift of the CoV_Y_. PEEP values for all subgroups (clinically set PEEP, EIT-PEEP, ARDSnet-PEEP) were higher in patients with fatal-outcome, with a significant difference calculated for the EIT-PEEP (*p* = 0.009).

### Comparison of the data with pediatric EIT studies

In the last two decades, several studies included EIT measurements for monitoring of invasive ventilated neonatal and pediatric patients. The feasibility and practicability of long-term EIT measurements in a large multicenter cohort of spontaneously breathing and invasive ventilated preterm and term neonates was recently published [23]. The benefit of EIT in children with pARDS was first evaluated more than 15 years ago [24, 25]. Wolf et al. described the effect of de-recruitment maneuvers (suctioning) in children with ALI/ARDS and how de-recruitment and regional ventilation loss, as well as loss of regional compliance can be monitored with EIT. In another study, Wolf et al. described the possibility of monitoring the effects of recruitment using the EIT technique and detection of compliance gain using the C_EIT_ approach as described by Costa [17]. Both studies provided the basis for the introduction of a personalized EIT guided monitoring strategy of invasive ventilated pediatric patients. Several years later the first data were published evaluating the personalized monitoring of regional ventilation in a pediatric cohort with pARDS using the EIT [26]. In this study, Rosemeier et al. concluded, that PEEP titration using the OD/LC approach (C_EIT_) resulted in a significant improvement of regional ventilation distribution and indicators of gas exchange. The preliminary findings of eight children with pARDS are in line with our findings and we could reproduce these findings in a larger set of patients, including both patients with nARDS/PLD and pARDS. Nevertheless, the study and PEEP titration protocol used in the above-mentioned study and our study differed slightly. Therefore, the data sets just can be compared with caution. Despite these promising findings, data regarding the advantage of EIT monitoring over ventilator-related monitoring are inconsistent. In a recent study comparing the C_EIT_ with C_DYN_ in a pediatric cohort of 12 children with pARDS (5 patients with severe ARDS and 7 with moderate ARDS), the authors revealed no significant changes between both methods of global compliance calculation, although the authors found a strong trend towards a better global compliance with the C_EIT_ approach, which still supports the findings of our study [27]. In the study provided by Inany et al. EIT was used to determine changes in regional ventilation using the CoV. The authors concluded that the CoV is a useful index to distinguish whether patients are prone to the evolution of gravity-depend atelectasis in the context of different modes of mechanical ventilation [28]. According to our data, the CoV seems to be a useful global parameter to determine regional changes in ventilation distribution towards gravity-dependent lung regions. When reducing the clinically set PEEP by -4mbar, the CoV_Y_ significantly decreased from each PEEP step to another. A decrease of CoV_Y_ from 0.5 towards 0.44 might indicate de-recruitment of ventilated lung area in gravity-dependent lung regions. But caution is required, as a decrease of the CoV_Y_ might also indicate a deflation of overdistended ventral lung regions in some patients, with a redistribution of ventilation towards the non-gravity-dependent regions.

### Comparison of the data with adult EIT studies and RCT trials

In recent RCTs in adult ARDS populations an individualized and personalized EIT guided PEEP titration was incorporated for the evaluation and comparison of the best EIT-PEEP strategy [12, 14]. In 2019 Zhao et al. revealed that an individual EIT guided ventilation strategy can optimize lung protective mechanical ventilation and might increase survival rates in adult ARDS patients [12]. In their prospective study the authors compared 24 patients with severe ARDS and an EIT guided PEEP finding strategy (Costa approach) with a matched historical control group of patients with severe ARDS (*n* = 31). The key findings were as follows: compared to the control group, the EIT guided strategy led to significantly higher PEEPs, higher global compliance, and lower driving pressures. Although non-significant, the survival rate in the EIT group tended to be improved (66% vs. 49%). In 2021 He et al. investigated an early (first 24 h) individualized PEEP finding strategy using EIT in an RCT with 117 ARDS patients (61 patients allocated to the EIT group and 56 to the control group using the low PEEP/FiO_2_ ARDSnet-table). The study showed a non-significant difference of 6% in the 28-days mortality (21% EIT group 27% control group) and a significant decrease of the ∆day 1 and ∆day 2 SOFA score in the EIT group, which was interpreted as a sign of improvement in organ function.

In a third RCT Hsu et al. confirmed these trends and findings and concluded that the EIT guided strategy using the Costa approach led to lower driving pressures in the EIT group and higher survival rates (69% vs. 44%) as compared to patients with PEEP identification based on the lower inflection + 2cmH2O of the quasi-static pressure-volume curve [29]. A major aim of EIT based research is the prevention of VILI. According to Becher et al. EIT is feasible to adjust PEEP to limit overdistension and alveolar cycling [14]. The group carried out a complex protocol in 20 ARDS patients to compare ARDSnet recommendations with their individualized EIT guided approach. By analyzing regional impedance changes in horizontal regions of interest, higher PEEP levels were detected, with better oxygenation and lower rates of alveolar cycling. These findings might indicate that EIT is capable to reduce OD and alveolar cycling, which were identified as main determinants of VILI [30].

The individualized EIT guided PEEP strategy has found influence in several reviews and meta-analysis, aiming to evaluate pros and cons of this promising approach [31–33].

In their systematic review Yu et al. 2023 summarized data sets of 8 RCTs using different EIT strategies and protocols for PEEP identification and found significant higher paO_2_/FiO_2_ ratios in patients receiving EIT guided strategies [32]. However, the meta-analysis failed to identify a benefit in respiratory system compliance when using EIT. In one of the most recent meta-analysis Songsanvorn et al. identified that EIT guided PEEP strategies led to higher lung compliance, lower mechanical power, and lower driving pressures, as compared to conventional strategies [33]. Most of the 13 included studies (62%) used the OD/LC approach as described by Costa et al. Both meta-analyses indicate that EIT has become a newly accepted monitoring technique. Nevertheless, data in the pediatric field are scarce and more prospective research is warranted in pediatric nARDS and pARDS patients.

### Future perspectives

The results and trends of the EIT studies conducted in the field of adult ARDS provide a strong basis for potential upcoming research in the pediatric populations. There are different barriers to overcome when facing nARDS and pARDS patients. The cause of nARDS and pARDS differ to a certain degree and both populations are only comparable to a certain level with adult ARDS patients. Besides the origin of ARDS and pathophysiology, body weights and lung volumes differ strongly in patients from 3 kg to 100 kg and lung capacities with lung compliance will evolve over time from the neonatal period to adult physiology. Ventilation strategies therefore differ in neonatal and pediatric patients when compared to adults, emphasizing that EIT studies in pediatric cohorts need to be well planned incorporating potential bias which might limit study power. To tie in with the call for more detailed and validating prospective studies our long-term aspirations are as follows: (a) plan a prospective RCT trial including longitudinal daily EIT guided PEEP identifications, compared to conventional PEEP finding strategies, (b) to compare EIT measurements with other recently established techniques as lung ultrasound score (LUS) to better correlate EIT data in nARDS and pARDS patients [34, 35], (c) using proteomic analysis to analyze and understand the involvement of down- and upregulation of biomarkers for the potential prediction of VILI in neonates and children.

### Limitations

Nevertheless, our study has several limitations. First, our EIT guided PEEP findings were carried out over short time periods (PEEP levels of 5 min). Long-term effects of PEEP could not be evaluated, and effects might be under- or overestimated. Therefore, improvements during the PEEP trial may represent only short-term effects, and the best ventilation strategy needs to be evaluated in a future trial over a longer period. Furthermore, using an approach with only five PEEP steps bears the risk of a missed identification of the adequate crossing-point with the Costa approach, when PEEP steps are not adequately adjusted around the crossing-point. The measured EIT-PEEP reflects only the PEEP to reduce the overdistension or lung collapse in the examined PEEP range. This needs to be considered when interpreting PEEP titration data. The more PEEP steps are included in the titration analysis, the more detailed information might be gained about the supposed optimal EIT-PEEP. Nevertheless, in all patients included in our analysis a crossing-point with a low and tolerable (≤ 5% loss of the lung pixels) overdistension or lung collapse could be detected. The timepoint when EIT guided PEEP titration was performed differed between patients (compare Table 1) and that might hamper the inter-individual comparison of EIT and ventilation data. Additionally, our study included patients with nARDS and pARDS with differing histories of disease progress. Therefore, our pooled data needs to be interpreted with caution, as higher inclusion rates and sample sizes of subgroups are highly warranted.

## Conclusion

An individualized EIT guided PEEP determination is feasible even in critical ill neonates and children with ARDS and might optimize regional ventilation distributions and lung mechanics. In both subgroups (nARDS/PLD and pARDS) PEEP level using the EIT-PEEP was identified to be lower than the clinically set PEEP as set by the attending physician. In both subgroups the lung compliance increased when adjusting PEEP towards the crossing point of the curve for the estimated OD and LC, but with decreasing values of the CoV_Y_, which might indicate a slight loss of regional ventilation in the gravity-dependent lung regions. We postulate that patients with nARDS and pARDS would benefit from a longitudinal EIT guided PEEP strategy, which might decrease incidence of VILI and might optimize survival rates in this population. However, for a final conclusion more research and RCTs need to be performed in neonatal and pediatric patients.

## Data Availability

All data generated or analyzed during this study are included in this published article [and its supplementary information files].
