# Correction: EIT guided evaluation of regional ventilation distributions in neonatal and pediatric ARDS: a prospective feasibility study

**DOI:** 10.1186/s12931-025-03292-9

**Published:** 2025-06-04

**Authors:** Leon Soltész, Judith Leyens, Marieke Vogel, Thomas Muders, Christian Putensen, Florian Kipfmueller, Till Dresbach, Andreas Mueller, Lukas Schroeder

**Affiliations:** 1 Department of Neonatology and Pediatric Intensive Care, University Children´s Hospital Bonn, Bonn, Germany; 2 Division of Neonatology, Department of Pediatric and Adolescent Medicine, University Hospital RWTH Aachen, Aachen, Germany; 3 Division of Neonatology, Department of Pediatrics, BC Women’s and Children’s Hospital, University of British Columbia, Vancouver, Canada; 4 Department of Anesthesiology and Operative Intensive Care Medicine, University Hospital Bonn, Bonn, Germany

**Correction to: Respiratory Research (2025) 26:60**

10.1186/s12931-025-03134-8

In the original publication of this article [1], the content of Table 4 was incorrectly published which is same as in Table 5. The revised content of Table 4 demonstrates the changes in dynamic lung compliance (Cdyn) and inspiratory as well as expiratory tidal volumes (TVi and TVe) during the PEEP titration. The corrected Table 4 is displayed below:

**Table 4:** All data are presented as median and interquartile range (25/75). Percentage change (∆) refers to the clinically set PEEP (baseline) compared with the according PEEP level. Abbreviations: C_dyn=_ dynamic compliance recorded by ICM Draeger or calculated $\:{\text{C}}_{\text{d}\text{y}\text{n}\:=}\frac{\text{T}\text{i}\text{d}\text{a}\text{l}\text{v}\text{o}\text{l}\text{u}\text{m}\text{e}}{\text{D}\text{r}\text{i}\text{v}\text{i}\text{n}\text{g}\text{p}\text{r}\text{e}\text{s}\text{s}\text{u}\text{e}}$), tvi = inspiratory tidal volume, tve = expiratory tidal volume

PEEP-Titration	C_dyn_ (ml/mbar/kg)	∆%	TVi (ml/kg)	∆%	TVe (ml/kg)	∆%
PEEP Baseline (BP)	0.5 (0.3–0.7)		6.7 (4.7–8.5)		6.3 (5-8.6)	
BP + 4 mbar	0.4 (0.3–0.7)	-20	6.3 (4.2–7.8)	-6	6 (3.7–7.8)	-4.8
BP + 2 mbar	0.4 (0.3–0.8)	-20	7 (4.4-9)	+ 4.4	6.6 (5-8.3)	+ 4.8
BP + 0 mbar	0.5 (0.3–0.8)	0	7.5 (5.1–9.3)	+ 12	6.4 (5.5–8.7)	+ 1.6
BP -2 mbar	0.6 (0.33–0.8)	+ 20	7.1 (5.6-9)	+ 6	6.9 (5.3–8.9)	+ 9.5
BP -4 mbar	0.6 (0.38–0.8)	+ 20	6.8 (5-8.6)	+ 1.5	7 (5.4–8.5)	+ 11

